# When prediction fails: a computational complexity view of stress

**DOI:** 10.3389/fvets.2026.1878636

**Published:** 2026-07-29

**Authors:** Sergey Budaev, Floriana Lai, Rachael Morgan, Ivar Rønnestad

**Affiliations:** Department of Biological Sciences, University of Bergen, Bergen, Norway

**Keywords:** anticipation, complexity, computation, control, homeostasis, prediction, stress, welfare

## Abstract

Living organisms are increasingly understood as predictive systems that anticipate near-future, fitness-relevant demands and proactively adjust physiology and behavior. Then, stress arises when environmental challenges exceed the range normally anticipated by the organism, eliciting stress responses that push regulatory systems toward or beyond their functional limits. These conditions are often associated with uncertainty, unpredictability and uncontrollability. We focus on the functioning of control systems when they fail to predict the environment and successfully control behavior. We hypothesize that under such conditions feedback controllers become increasingly obsolete and can be dynamically discounted, providing a fitness benefit. Computational complexity of adaptive control is expected to reduce the size of the controller program, the complexity of its output and therefore the complexity of adaptive behavior. We briefly review the literature suggesting that reduced complexity of behavior may serve as an indicator of developing stress. Finally, we outline several approaches and R software packages for the measurement of complexity.

## 1 Introduction

Life occurs in changing, uncertain, risky, dangerous and often harsh environments. All organisms, from simplest bacteria to our own species, constantly face different challenges that elicit stress, which is a fundamental evolved mechanism to cope with a broad range of threats and risks. Stress is closely linked with the important issues of health, welfare and wellbeing (1–3). Even though some degree of stress is an integral part of life, repeated, acute or prolonged stress can be detrimental (2–4). The organism usually responds to a demanding challenge with a cascade of adaptive regulatory processes: physiological, cognitive and behavioral reactions directed at coping with the challenge, including threat appraisal, maintenance of the homeostasis and recovery from possible damage (5, 6). In addition to the *stress response*, one can also distinguish the *state of stress*: an internal condition of the organism characterized by reduced capacity for adaptive control. For example, the organism can display an adaptive response to a stressful challenge (stress response) without being in the state of stress or, if the stress response was sufficient to cope with the challenge, without subsequently transiting to the state of stress. This view is similar but not identical to the concept of distress^1^, defined as “being stressed” (2) or as a biological state in which the stress response exerts a harmful effect on welfare and health (7, 8) or when the challenge is subjectively perceived as unpleasant (1, 3).

While originally viewed as a non-specific physiological response to noxious stimuli, stress is now typically framed in terms of uncertainty, unpredictability and uncontrollability (9, 10). Uncertainty about the future, including the consequences of the animal’s own choices, is recognized as a fundamental challenge faced by most organisms. To survive and reproduce, animals benefit from efficient strategies that reduce uncertainties about their environment and the likely results of their actions (10, 11). The organism therefore predicts its near-future fitness-related needs and adjusts its functioning to prepare for the anticipated challenges (12–14). This emphasizes the first-person Umwelt view of the organism in its world (15–18).

In this paper, we outline a broad theoretical approach for understanding integral system-based stress in animals based on prediction and complexity of behavioral action control. Specifically, we focus on the behavioral activity that arises when an organism comes to the state of stress, i.e., fails to accurately predict and control its environment, resulting in excessive prediction error. For example, large literature evidenced that unpredictable and unavoidable stress is significantly more detrimental than predictable in various species [e.g., (19–21)]. We argue that the state of stress is strongly reflected in the overall complexity of behavior: the pattern of actions and interactions with the environment. We briefly review the literature showing that reduced overall complexity of behavior can provide an indicator for developing stress and point to possible metrics. We also review the existing R software libraries for quantifying complexity. Box 1 presents the definitions for basic terms.

Box 1 **Glossary**

*Stress:* an adaptive regulatory mechanism evolved to cope with threats, risks and challenges, environmental or internal. Stress can refer to a demanding or challenging *condition*, an aroused *state* of the organism, as well as a cascade of adaptive physiological, cognitive and behavioral *responses* directed to threat appraisal, maintenance of the homeostasis and recovery from the possible damage. For example, physiological responses to stress usually include activation of the sympathetic nervous system, HPA axis, release of adrenaline and cortisol. These responses may bring about beneficial or harmful consequences depending on their magnitude, duration, context, the state (condition) of the organism and other factors. Stress can involve a short-term challenge (*acute stress)*, or long-lasting or repeated challenge (*chronic stress)*. Stress is characterized by three crucial elements: it is aversive, involves general activation of the organism, and is associated with unpredictability and uncontrollability.

*Stress response* versus *stress state*. We distinguish stress *response*, i.e., an adaptive response to an internal or external challenge and stress *state*, i.e., an internal condition of the organism characterized by a reduced capacity for adaptive control. An organism could display an adaptive response to a stressor without being in the state of stress. Such a response could diminish and eliminate the challenge; otherwise, the challenge could exceed the adaptive capacities of the organism, putting it to the state of stress and further to distress: unpleasant subjective feeling, deleterious welfare and health consequences.

*Stressor:* An internal or environmental factor that poses an adaptive challenge or demand on the organism. Stressor requires an adaptive regulatory response from the organism, otherwise it disrupts homeostatic balance leading to the stress state.

*Regulation*: a coordinated set of physiological, cognitive and behavioral mechanisms and processes that control, adjust, accommodate and constrain the adaptive functioning of the organism, promoting successful survival and reproduction in a dynamic, changing environment.

*Computational and adaptive capacity:* The upper limit on the ability of a system to perform (or keep) a specific function. *Computational (information processing) capacity* refers to the maximum performance (e.g., operations per unit time) of a computational system. *Regulatory (adaptive) capacity* is the system’s ability to adjust to the operating conditions via its regulatory (e.g., feedback) mechanisms to keep a specific level of performance. Regulatory and computational capacity can scale up and down depending on available energy and other resources. For example, when the resources are insufficient, the maximum possible level of performance is reduced; if a higher level is required, it cannot be reached within a time constraint. For an organism, reduced adaptive capacity under acute, repeated or persistent challenge (i.e., reduced *resilience*), results in exhaustion, fatigue and physiological breakdown. Adaptive capacity is distinct from adaptive *capability*, meaning whether an adaptive task is achievable as the organism approaches the limit of its adaptive capacity to maintain the homeostasis, without respect to the specific capacity level.

*Homeostasis*: a set of regulatory process that function to maintain the stability of the internal environment to ensure its optimal performance even in presence of significant external disturbances.

*Allostasis*: a set of regulatory processes by which an organism achieves adaptive functioning dynamically through change. It is based on predictive regulation of physiological responses, energy balance, emotion, cognition and behavior. Essentially, the organism anticipates an incoming challenge and executes preparatory responses, resulting in effective pre-emptive adaptation. However, such responses carry significant metabolic costs which can accumulate as *allostatic load*.

*Turing machine*: an abstract model of computation based on symbol manipulation following specific rules. It includes an infinite tape with discrete cells containing symbols, a head that can read one symbol and then, depending on it and the machine state, write one symbol. Then, the head moves forward or backward along the tape, or halts. A function is computable if there is a set of instructions that result in completion of the function computation. In the general case, the computation is performed regardless of the number of time steps required to compute.

*Tractability*: in a broad usage means “practical feasibility.” In theoretical computer science, tractability is framed in terms of the time and other resources (e.g., memory, program size) required to complete a computation task. A problem that cannot be solved within a sufficiently limited resources is called *intractable*. Tractability usually indicates how “scalable” the algorithm is for inputs of increasing size. Tractable then denotes a problem solvable in a polynomial time of its input size.

*Complexity classes*: basic sets of decision problems grouped according to the computational resources required to solve them. It is common to distinguish the following complexity classes: **P**: problems are decidable and solvable within a polynomial resource; **NP**: problems are intractable but can be verified in polynomial time; **NP-hard:** the class of problems at least as hard as the hardest problems in NP, can be undecidable; **NP-complete**: the hardest problems in NP; widely considered effectively intractable.

*Kolmogorov (algorithmic) complexity*: is a quantity defined for a finite data (symbols) string and refers to the shortest algorithm running on a Turing machine that outputs the data and halts. A simple regular string like “100,100,100,100…100” (even if its length is very large) requires a small program to generate and is said to have low complexity. By contrast, a stochastic, algorithmically random, string requires an algorithm that cannot be smaller than the string itself, it has no shorter description and is said to be incompressible.

## 2 Prediction, control and stress

At the level of the individual cell, stress refers to harmful environmental effects that disrupt the cellular homeostasis, accumulating imbalance, damage and ultimately shaping the organism’s response to the challenge (22, 23). Because cellular decisions are primarily modulated by the energy status (24, 25), cellular stress response involves maintenance of ATP (the primary energy carrier) at the level required by the most essential processes (26).

Evolutionarily more advanced organisms, especially animals with a developed nervous system, respond to challenges through anticipation and prediction in addition to direct response to an immediate challenge (12, 13, 27). For example, when a visual cue predicted a stressor one minute beforehand in European sea bass (*Dicentrarchus labrax*), the fish showed reduced behavioral reactivity, lower cortisol release, and altered gene expression indicative of neuronal activity in the telencephalon, suggesting that the response was modulated by anticipation-based appraisal (28). Also, omission of expected reward excited frustrative aggression without cortisol response in the Atlantic salmon (*Salmo salar*) (29). A similar pattern was documented in the rainbow trout (*Oncorhynchus mykiss*) with smaller fish characterized by a stronger frustrative response to failed expectation (30).

Several related theoretical frameworks have been proposed to understand stress at the level of the whole organism, emphasizing anticipation, preparation, and control in challenging situations. One of the most prominent anticipation-based and integrative frameworks for analyzing stress is *allostasis* (31–35). Allostasis is an extension of the concept of *homeostasis*, which describes mechanisms that maintain the stability of the internal environment. In contrast, allostasis emphasizes *predictive regulation* of physiological functions and energy budgets, enabling organisms to maintain functional stability through change.

The nervous system, brain, and cognitive processes constitute core elements of the organism’s predictive architecture (35), operating in close coordination with endocrine systems to support anticipatory regulation and stress responses (36, 37). In allostasis the organism does not maintain a single, fixed optimal steady state, but engages in flexible, adaptive regulation, so that the homeostatic set-points are changing depending on the anticipated challenge and the predicted need. Allostasis involves a coordinated regulation including brain and body, cognition and endocrine systems, rather than purely isolated feedback mechanisms. As a consequence, different stressors that occur in different contexts and act on the organism in different states can result in different patterns of activation of the sympathetic nervous and adrenomedullary hormonal systems (32, 36).

The challenges that an animal meets can elicit preparatory allostatic responses, but they always come at metabolic cost. Such cost, *allostatic load*, may become detrimental when challenges are inaccurately predicted, exceed organismal capacity, or persist chronically (34, 37). Mild to moderate stress can already change energy allocation. For example, in many organisms moderate stress results in reduced growth, reflecting adaptive prioritisation of maintenance over anabolic processes (38, 39). Persistent or extreme allostatic demand exceeding regulatory capacity may trigger an emergency life-history stage characterized by a re-prioritization of energy toward immediate survival (40, 41). Beyond this extreme point, failure of regulatory control can ultimately result in death. There is thus a paradox, allostasis and stress have evolved to adapt organism to challenges in dynamic environments but can also lead to apparently maladaptive outcomes (5, 14, 42, 43).

The central role of uncertainty and prediction-based cognition has been emphasized in the Bayesian brain framework (44–49) applied to stress (10, 50, 51). Here, the organism living in a variable environment is primarily confronted with the fundamental task of minimizing uncertainty, which is quantified as the information-theoretic “*free energy,*” a common function for both perception and action (47, 52, 53). This can occur either (a) by updating the brain’s internal model—probabilistic belief—of the environment to fit with the sensory data or through *active inference*, including actions that can give better sensory information to update the internal model or (b) behavioral action that moves the organism into an environment that better agrees with its pre-existing internal model, so a model update is not needed (46, 47, 52). For example, avoidance learning in zebrafish depends on two neural ensembles in the dorsal telencephalon: one encoding the primary cue-based rule (“the punished color is dangerous,” “the unpunished color is safe”) and the second ensemble that represents the prediction error: the discrepancy between the predicted visual sensation of scene flow (as the fish swims) and the actual input from the visual system. The second ensemble allows the fish to act efficiently through minimizing prediction error (54).

While initially developed in view of rather advanced cognitive systems that are able to calculate, represent and use probabilities as well as accumulate information about the different choice options, the active inference paradigm is being extended to non-neural and non-cognitive processes such as morphogenesis (55), elementary chemical oscillators (56) and the origin of life (57). Minimizing the free energy is computationally and energetically costly (58, 59). An organism unable to reduce the informational free energy finds itself persistently in a high uncertainty state irrespective of its actions. This imposes sustained energetic costs, burdening the allostatic overload and bringing about a range of adverse effects and resulting in systemic pathology (10, 51). To prevent detrimental consequences, the organism is predicted to alter the goal state of its cognitive system, transitioning toward habituation in response to sustained environmental adversity (10). Stress is regulated by neuromodulatory, neuroendocrine and immune systems that act on different time scales. Sustained unpredictability and uncontrollability, pointing to failed habituation, bring about a heightened neuroendocrine response and generally shift the organism’s control of behavior from goal-directed to habitual, routine-based (50, 51, 60). For example, glucocorticoid receptor blockade, preventing recovery after stress, resulted in more frequent failures in the detour task test than in control in a cichlid fish *Neolamprologus pulcher* caused by inflexible, routinized response (61). Similarly, acute exposure to the alarm substance induced repetitive responding of zebrafish in a Y-maze with a tendency to increase over time independent of the action outcome (62).

The control systems perspective (11, 63–65) views stress as a consequence of failed control of external or internal fitness-critical variables. This perspective is based on the classical theory of regulatory control (66–68) that has many applications in industrial automation (66, 68), functioning of biochemical networks (69, 70), behavioral control (71) and general design of biological systems (72–74). This perspective implicates interconnected and hierarchically nested feedback (reactive, error-correcting) and feed-forward (anticipatory, predictive) mechanisms. Closed-loop feedback regulation is based on maintaining a set-point, which is usually computationally simple, allowing effective self-correction even in cases of pronounced unanticipated disturbances. In contrast, open-loop feed-forward regulation requires an accurate internal model. Biological feedback controllers usually work in concert with feed-forward, reporting prediction error to model-based control. While the distinction between feedback and feed-forward control is a simple way of thinking about adaptive control, live organisms are characterized by an intricate, multilayered combination of both types, they are “deeply overwired” to facilitate robust adaptation in dynamic environments (73, 74), similar to complex engineering systems (75).

All regulatory systems are constrained by fundamental trade-offs, such as robustness versus flexibility (11, 67, 72), explaining why more robust controllers have lower plasticity (11, 73, 74). For example, an adaptive feedback-based control system should respond rapidly to a perturbation with a high signal gain towards the set point. However, such strong and rapid response to error could cause an overshot over the set point and, if further overshot-correcting actions are also strong, maladaptive fluctuations (67). Increasing flexibility, gain and complexity might therefore improve performance on more regular tasks but reduce stability under disturbance uncertainty, delays or non-foreseen dynamics. On the other hand, limiting control signal gain, responsiveness and flexibility would improve the system stability margins, i.e., robustness (67, 73).

One of the most common feedback control paradigms is proportional-integral-derivative (PID) often used in industrial automation (66–68). PID control involves robust corrections of the disturbance through three components that work on the instantaneous deviation, past control errors as well as immediately predicted error: (a) the correction proportional to the magnitude of the deviation from the set point: **P** (i.e., a small correction for a small error and large for a large error), (b) the cumulative sum of past errors correcting the residual error over time: **I**, (c) a component anticipating the next error that is proportional to the rate of change for the error, its derivative: **D** (mitigating overshoot and providing additional stability under varying and rapid disturbances). PID provides a viable model for genetic and molecular regulation (76–78). It has been used to model human perceptual and sensorimotor control (71) and is expected to work in computational psychiatry for arousal and affective control (79).

Generally, a challenge shifting some controlled process results in a discrepancy detection and the feedback acting to nullify it. Then, various control errors are possible. The system can overrespond to the challenge if the **P**, **I** or **D** component parameters are not precisely adjusted. For example, if the **P** gain is too high, the system responds too sharply to the disturbance. If **I** gain is too high, the response eliminates past errors too rapidly. This will bring about a deviation between the set point and the process persisting after the challenge has gone (66, 68). Human-designed systems are often tuned to keep the process variable precise. Unlike this, for living organisms, misalignment of control parameters to overrespond and exaggerate defensive action would avert the more life threatening errors and therefore result in higher fitness in a stochastic environment (80–83). This may explain *hormesis*, a biphasic response pattern when a weaker challenge exerts a stimulatory effect while high challenge, an adverse effect (84–86). Hormesis therefore involves an overcorrection (11, 87) or overcompensation (84) to a homeostatic challenge which, if the initial intervention proves unsuccessful, can further escalate to uncontrollable stress condition.

Stress responses are triggered by perceptions of unpredictability and uncontrollability, based on measuring a discrepancy between the anticipated value of the fitness-critical variable and its actual state. Large or persistent discrepancies indicate failure to control. Furthermore, the strongest level of stress response is especially likely to emerge following unexpected loss of control, when formerly predictable conditions abruptly change. Uncontrollability can be inferred from delayed or incomplete physiological recovery following an environmental challenge, while unpredictability results in a lack of anticipatory responses. Thus, uncontrollability corresponds to the inability of feedback mechanisms to maintain physiological variables within adaptive target ranges, while unpredictability arises from failures of feed-forward processes to anticipate upcoming challenges. Predictability and control depend on both the environment and the organism, so control failures and stress can be caused by external environmental effects as well as internal causal factors (11, 64). When a failure to control a fitness-critical variable is detected from a large and growing prediction error, the resulting stress responses may elicit modification of the model used by the feed-forward controller or other adaptive mitigating behavior change. This links the control system perspective with the active inference paradigm (11). The classical mechanistic control theory (proportional-integral-derivative, PID) can be considered as a linear approximation for the Bayesian free energy minimization (88).

The adaptive calibration model of stress (89) postulates that the autonomic nervous system and neuroendocrine stress response systems have evolved to control conditional adaptations that regulate individual life history strategies and developmental trajectories. Activation of autonomic and neuroendocrine responses early in life provides information about the general characteristics of the environment, including its predictability, typical challenges, risks, and affordances. Over development, this information becomes embedded in the parameters—set points and reactivity patterns—of these physiological systems. Consequently, repeated stress-related activation during development supplies signals that tune both physiological and cognitive systems. These signals convey probabilistic information about the environment, calibrating the internal predictive model that is involved in feed-forward model-based adaptive actions (11, 89). An illustration of the crucial fine-tuning role played by early stressors is the recent study on the (anadromous) Atlantic salmon: development under stable conditions during the early freshwater phase leads to poorer robustness, health and slower growth in sea cages (90). Early inescapable stress in salmon mitigates the serotonergic responses to acute stress later in life (91). Similarly, fruit bats that have experienced frequent environmental changes during early development are subsequently bolder, more flexible and efficient in response to novel challenges in the wild (92). This fits with a more general body of evidence showing that environmental enrichment promotes behavioral flexibility and neural plasticity (93), improving welfare (94, 95).

## 3 Complexity

From the previous section it is clear that stress can result as a consequence of prediction and control *complexity*, which in more extreme cases is a *failure to predict and control*. But what is complexity in the first place? Complexity is a general concept that partly lies “in the eye of the beholder” (96–98). For example, there is no universal definition of a complex system (97). A widely held view is that a system is considered “complex” when it is difficult to describe, understand and model: “a system is not complex by some abstract criterion but because it is intrinsically hard to model, no matter which mathematical means are used” [(99), p. 6]. Some essential characteristics of complexity include high dimensionality, interactions between multiple networked elements, and nonlinearity leading to reduced predictability and uncertainty about causal relationships (99, 100). A prominent characteristic of complexity is linked with the susceptibility of the system to end up in uncertainty (98). Lloyd (101) distinguished three aspects of system complexity that may be fundamentally distinct: (a) how hard is it to describe? (b) how hard is it to put together, create? (3) what is the degree of organization? For example, a system could be simple to create (by the first person) yet hard to describe and predict from a third person perspective. From this standpoint, organisms, not developed by us with complete knowledge of the design, are complex systems, characterized by intricate evolved multi-layer organization hard to describe and model at all levels (102–104). From the organism’s own perspective (16, 18), the environment is complex whenever there is no easy and straightforward way to describe, understand and learn from it, put together a predictive model, select an adaptive policy and plan goal-directed actions (16, 105). There is a close link between complexity and intelligence: a complex, dynamically changing environment putting the organism into novel, challenging and risky situations requires an ability to learn from previous experiences, predict future threats, choose better options and flexibly exploit opening affordances (106–108). Solving local and immediate problems as well as planning remote future actions require intelligence (105). The importance of environmental complexity, eliciting neural and further behavioral complexity, in turn, determining animal welfare has been acknowledged decades ago (109). The modern welfare frameworks emphasize the opportunities for engaging in varied, complex experiences that contribute to the acquisition of competences leading to flourishing (110).

At an abstract level, the notion of complexity can be reduced to *difficulty to compute*. Theoretical computer science has developed fundamental mathematical formalization for complexity of algorithmic calculations performed with deterministic Turing machines (111, 112). First, there are problems that cannot be solved for all cases by any possible algorithm whatsoever: *undecidable* problems (113, 114). For *decidable* problems, there exists an algorithm that can decide any case of the problem. Yet, the amount of resources needed to solve such a problem can vary enormously, making many decidable problems unfeasible in practice. Computational complexity measures the algorithm performance in terms of resources, such as execution time, the number of the algorithm steps or the memory required, depending on the size of the input data. The upper and lower bounds of the algorithm resources are described in terms of the Landau’s “big *O*” (i.e., worst case) and “big *Ω*” (i.e., best case) notations. These indicate how “scalable” the algorithm is for inputs of increasing size. It is common to distinguish the following categories of growing complexity: *O*(1) constant time, *O*(*log n*) logarithmic time, *O*(*n*) linear time, *O*(*n^p^*) polynomial time, *O*(*k^n^*) exponential time and *O*(*n*!) factorial time. Most of the algorithms that are used in practical computations (searching, sorting, finding the shortest path in an undirected graph etc.) are upper bounded by a polynomial of the input size. Such computational problems are considered *tractable*. The complexity class of all decision problems that are solvable by a Turing machine in polynomial time of the input size is denoted by ***P***. These include problems that can be efficiently solved within a reasonable amount of computational resources. To define a further class of complexity, one need to implicate the concept of a certifier, that does not produce a computational solution but verifies that the solution is correct. Then the ***NP*** (nondeterministic polynomial) class consists of decision problems for which a solution can be verified in polynomial time even though no efficient algorithm is known that solves the problem. In other words, ***NP*** problems are *intractable*, i.e., unlikely solved within a reasonable amount of resources. Further, ***NP*-hard** problems are at least as hard as the hardest problems in ***NP***, even though not all ***NP*-hard** problems are in ***NP***. ***NP*-hard** problems can be undecidable. Finally, the class of the hardest problems in ***NP*** makes the so-called ***NP*-complete** class of problems. There exist an algorithm to solve an ***NP*-complete** problem, but it is far too prohibitive. An interesting property of ***NP*-complete** problems is that all such problems are equivalently hard and can be reduced to one another in polynomial time.

Even though no efficient polynomial-time algorithms are known for ***NP*-hard** or ***NP*-complete** problems, it may in many cases be possible to cope with the complexity by approximation algorithms (providing solutions approximating the optimal ones with a guaranteed performance ratio), heuristic algorithms (with no guarantee of optimal solutions, but sufficiently close to it for some practical uses, e.g., the genetic algorithm or simulated annealing), as well as special cases when ***NP*-complete** problems turn to polynomial for certain restricted classes of inputs or for specific parameters (i.e., even if input is large, the problem remains tractable for a specific restricted parameter set).

Dynamical processes can be hard to predict. There is an intrinsic limitation to prediction either because of chaotic dynamics or large number of effective degrees of freedom (115). Biological feedback control loops include molecular signaling cascades involving multiple chemical events where it is extremely hard to diminish noise and error (116). Therefore, many if not most biological and especially cognitive problems (essentially predicting the future and the best action or policy) occurring in complex changing environments can be demonstrated to be computationally intractable (117–120). Further, systems (biochemical, neural, and cognitive) implementing self-reference can be undecidable (121). The classical examples of problems characterized by computational intractability are protein folding (122, 123), gene regulatory networks (124, 125), evolutionary fitness landscape optimization (126, 127), visual perception (128–130) and behavioral decision-making (118, 131, 132). In spite of the extraordinary computational challenges, biological systems apparently have found good enough satisficing solutions through evolved approximate, heuristic algorithms and architectural constraints (16, 119). For example, the multiple layers of computations executed by biochemical, genetic and neural systems allows to take advantage of affordances provided at the different levels and scales—from microscopic to macroscopic—effectively splitting the larger problem into a series of smaller approximate problems and generalizing the concept of “hacking” (133).

This aligns with a general metaphor of the biological organism as a computer—or a collection of computers at the different levels—from genes, regulatory networks to neural systems and cognition: all biological systems can be understood as computing machines (134–136). As such, all living systems and biological processes are subject to computational complexity constraints (102, 137–140).

## 4 Living organism as Turing computer

The idea that the organism can be thought of as a computer, or an assembly of computers working at different levels (genetic, biochemical, hormonal, neural, cognitive) that adapt to its environments through computation, prediction and adaptive action must not be understood too literally. Most human-made electronic computation machines are designed and built according to the standard von Neumann architecture (141) that includes (a) the central processing unit (CPU) with the arithmetic unit and controller for program execution, as well as (b) read-write memory, (c) input and (d) output devices. Biological living systems are very different from this simple design. An abstract Turing machine is a deterministic symbol manipulation automata with a finite set [usually two, (142)] of possible states (143, 144). As in the Turing computation abstraction, biological systems have definite internal states, these states can change in response to signals from the external and the internal environment, executing state transitions. Further, biological systems can emulate reading and writing symbols, the most iconic example is DNA/RNA. The foundation of all life processes subsists in autocatalytic chemical reactions (104, 145). Most known kinds of abstract computing automata can be implemented in out-of-equilibrium oscillating chemical reactions (146–148).

Computation is typically viewed as a human activity that involves specially designed electronic, chemical or quantum tools. In contrast to this, a wide range of physical systems can be legitimately viewed as intrinsically computational provided they include (“implement”) a mechanism that functions to manipulate some physical (but medium-independent, i.e., defined solely in terms of degrees of freedom) vehicle based on differences between specific components or units of such vehicle according to rules (149). Computation can therefore be objective, concrete, physical and ubiquitous: computation can be found everywhere (144, 149). More generally, abstract computation can be defined in the physical domain as representational activity that allows a physical system to predict an abstract evolution through a computing cycle (150, 151). Therefore, biological systems are genuinely and intrinsically computing (152). The necessary condition for computation is sufficiently high degrees of freedom in the physical system, producing arbitrary representation: physical to abstract mapping, and instantiation: abstract to physical mapping (152). Thus, the thesis that information-processing mechanisms in all living organisms can be viewed as biological computers in an abstract sense would not be surprising or counterintuitive (134, 136).

## 5 Prediction, algorithmic complexity and stress

We can proceed by modelling an organism as a universal Turing machine ***T***. The organism interacts with its environment via regulatory actions and behavior. We further assume that the behavior is produced by a controller program ***P*** that is run by the organism. It is reasonable to surmise that this program gets the input from the environment via the sensory system, calculates the adaptive responses to these inputs (in the simplest case it can work as a lookup table based on “if-then” logical branching) and outputs them into the outside world as behavior. Thus, ***T*** processes sensory and internal state inputs, produces action outputs and updates control variables. The behavior of the organism can be framed as a data string ***b*** that is produced as the output of the program ***P*** that runs on ***T***. The complexity of the ***b*** is determined by the length of the shortest program ***P*** that produces ***b*** and then halts (153):


K=min(∣P∣:T(P)=b)

This defines the concept of algorithmic complexity for ***b*** (154–156). The invariance theorem (153, 157) determines that a range of different Turing machines will produce asymptotically equivalent results that will differ only in an additive constant unrelated to ***b***. The effects of the different machines ***T*** on sufficiently long ***b*** outputs are therefore bounded. Then, a short ***P*** will produce a ***b*** with low complexity while a long ***P*** will generate a ***b*** of high complexity. In a general case, the upper limit for the complexity of ***b*** is set by a pattern indistinguishable from random produced by a very long ***P***. Here is the principal point: a data pattern that is hard or impossible to describe in a way shorter than itself—that cannot be compressed—is deemed prohibitively complex. So a random pattern that cannot be meaningfully compressed, described, modelled, predicted and controlled is deemed intractably complex. Unfortunately, the value of the algorithmic complexity ***K*** is in general case uncomputable: there exist no algorithm that computes its exact value (153). However, approximations are available for short data strings and their combinations using the block decomposition method for an upper bound of ***K*** (156–158) (illustrated in Box 2).

Box 2 **Illustration of data pattern complexity**

To illustrate the concept of complexity we can take two short data sets.

•Regular (simple pattern): 1 0 1 0 1 0 1 0 1 0 1 0 1 0 1 0

•Irregular (complex pattern): 1 0 0 0 1 0 1 1 0 0 1 1 1 1 0 0.

Both have the same variability expressed in standard deviation: sd = 0.52.

•*Kolmogorov (algorithmic) complexity*: Regular data is characterized by lower *K*:

Regular: *K* = 10.26

Irregular: *K* = 33.12.

(note that its absolute scale of *K* is arbitrary and depends on the details of the approximation algorithm)

•*Permutation entropy:* Regular data is characterized by lower permutation entropy:

Regular: *H* = 0.11

Irregular: *H* = 0.36.

There is an intrinsic link between complexity, computability, “compressibility,” predictability and controllability. An exceedingly complex environment requires a big optimal controller program involving a large amount of computational resources that will yield a complex adaptive behavioral output (***b*** string). This results in a high ***K***. Living organisms always have limited computational resources, basically restricted by the energetic costs and the net rate of energy input. This means that there is always a hard limit on the complexity of the algorithm—the “*O* limit,” e.g. *O*(1), *O*(*n*) or simpler *O*(*n^p^*)—and therefore the size of the program ***P***. As with human-designed physical computers, there are strong limitations on the materials needed to build the computer (e.g., accretion of neural tissue), engineering effort needed to design and build it (evolutionary time, learning time) and the energy required to operate the system (e.g., energy assimilated from food).

What happens when the computational challenge given by the environmental complexity exceeds the computational capacity of the organism? Unlike a human-designed software that just fails or crashes, living organisms have evolved through the natural selection. Therefore, we can expect that a smaller program ***P′***, analogous to “lossy compression” (i.e., with some information lost in the process) making use of evolved approximations or unique heuristics arising from serendipity of mutation and selection could happen to be “good enough” to win the competition with conspecifics, i.e., successful survival and reproduction. Yet, there is no guarantee that such approximate “lossy compression” solution is sufficiently good in different contexts, nor indeed that such a solution will ever evolve. Organisms can die as a result of computational failures with the genes that contribute to such failures being lost.

**Figure 1:**
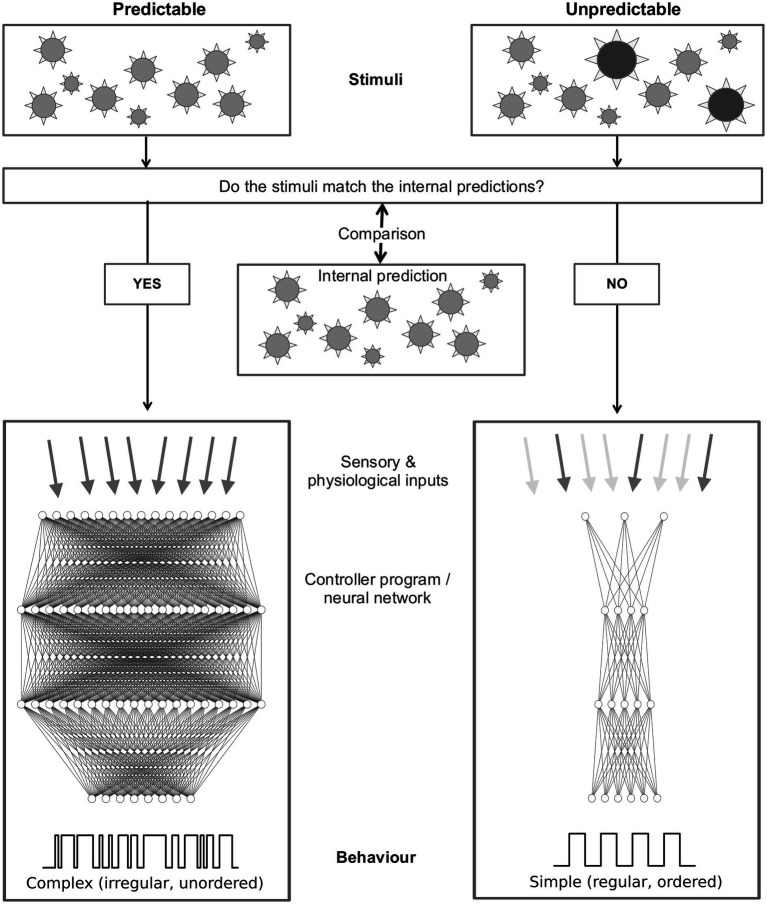
An illustration of dynamic pruning of feedback controller program under conditions of unpredictability and uncontrollability that result in simple regular behavior output. Note that we use “regular” in the mathematical sense, to describe a pattern, meaning “simple,” “ordered,” “repeatable,” “compressible.” Regular or irregular in this sense has no link with a biological regulation, (dys)function, disorder or pathology.

When no efficient adaptive controller program that meets the computational demand of the environment exists, the organism finds itself in a situation of unpredictability and uncontrollability. If no program ***P*** or ***P′*** could cope with the environment complexity, the only adaptive options are to escape away from the unfortunate environment or to wait until the conditions become more favorable. Both these options should not depend on accurate adjustment to the environment, since collecting precise inputs from the environment has already been established unfeasible. This means that many or most environmental inputs should be ignored. In other words, the only option for the organism in such a situation is to switch to a rigid active or passive policy with limited feedback from the environmental signals. Limited input translates to a smaller number of feedback control mechanisms, limited size of the controller program ***P*** and further to a lower complexity of its output ***b*** (Figure 1). The overall result is the reduction of ***K***. One can further expect frugal and economic programs to provide better chances to survive and reproduce. Whenever the organism chooses an active or passive strategy, it must save its resources for an uncertain yet possibly (or hopefully) better future. Information processing in biological systems ultimately translates to the available energy resources (24, 25). Running a small program that requires less computational resources would incur lower cost and improve fitness.

In a general case, the computational capacity of the organism can reach a hard cap in two ways. First, an unanticipated surge of environmental complexity, such as sudden events, can exceed the “*O* limit” of the information processing system. Second, given a particular environment, the information processing capacity of the organism can be impaired if its energetic resources are exhausted, or if the environmental challenge exerts a direct detrimental effect on the information processing system, such as endocrine disruption, hypoxia or toxic exposure. Both ways would result in loss of predictability and control, that is, the stress condition.

Essentially, stress can be thought to occur when the “standard” controller program containing numerous complex input–output mappings performs badly. Many of the inputs and feedback controls become obsolete and can be dynamically trimmed off along with the related program modules without detrimental consequences or even with significant energy salvage. In the evolutionary perspective, higher computational complexity could be tolerated or even maintained when it improves fitness, supporting more precise and efficient adaptive control for flexible behavior (105, 108, 159–161). But whenever prediction and control fail, trimming an obsolete, costly and non-functional computational machinery will likely improve fitness. Thus, stress brought about by excessive computational complexity and intractability of adaptive control is expected to reduce the size of the controller program and the complexity of its output: adaptive behavior.

## 6 What do empirical studies say?

It has long been known that the development of stereotypic behavior is a reliable indicator of stress and poor wellbeing in animals (162). Apart from such severe phenomenon as stereotypes, an array of studies demonstrated that milder stress can indeed result in reduced overall behavioral complexity in a variety of animal species (see also 109). For example, locomotor complexity was reduced in a dose-dependent manner in zebrafish injected with acetic acid (163). In the sea bass, invasive tagging and water contamination with methylmercury increasingly reduced the complexity of locomotion determined via optical flow analysis of video (164). The complexity of locomotor behavioral sequences in chicks decreased with stress caused by food limitation (165). Female Spanish ibex displayed reduced complexity of exploratory behavior as a result of stress caused by pregnancy and parasitism (166). Similarly, parasite-infected sheep show lower complexity of activity sequences (167). Bottlenose dolphins exhibit reduced behavioral complexity in presence of fishing and power motor-boats but not kayaks, presumably as a result of pronounced disturbance stress (168). Dolphins living in environments characterized by higher level of anthropogenic pressure show a lower behavioral complexity (169). The same pattern was found in killer whales, southern right whales and grey seals: anthropogenic stress is linked with reduced behavioral complexity (170). Yellow-breasted capuchins living in a low stress zoobotanical garden were characterized by higher complexity of behavioral sequences than animals from wildlife rescue center with poor conditions (171). Japanese macaques infected with a nodular worm, impaired health, low dominance and ageing also displayed lower levels of locomotory and foraging complexity (172). A similar pattern with thermal stress reducing complexity, is also observed in the *C. elegans* nematode (173). In humans, stress brings about emotional fragility, where transition towards depression is accompanied by increasing autocorrelations and cross-correlations between mood states, i.e., higher predictability and lower complexity (174). Similarly, heart rate variability tends to be lower in patients with depression and other psychiatric disorders (175). Finally, decreased complexity as a consequence of arousal and stress has also been suggested in computational models (17).

Thus, there seem to be a consistent pattern in a variety of species: stress is linked with reduced behavioral complexity. These results are often observed at the high level, the behavior of the whole organism, which allows to use complexity as a highly sensitive and non-invasive indicator of welfare and wellbeing (109, 176). This aligns with a common assumption that a restricted behavioral repertoire arises from limited environmental affordances and therefore points to reduced welfare status (177). To the extent uncertainty and unpredictability is implicated, pruning of ineffective control systems is expected to bring about a reduced behavioral complexity even in behaviors that are not directly compromised by curtailed affordances. The crucial point from this perspective is that the unit of analysis should be possible to model as an integral computational system. We would not necessarily expect a similar link between stress and complexity of any given isolated variable or index.

The relationship between stress and integral behavioral complexity suggests an interesting applied perspectives. Obviously, such a measure would be well suited for continuos automated monitoring, when unexpected deviation from an established pattern could serve as an early signal (a “red flag”) of developing stress, perhaps not yet easily detectable at the physiological level. This would be further enhanced through the use of computational models—simulation or machine-learning—that could provide predictions on the likely future stress development and the resulting wellbeing and health effects. For example, digital twins could run in parallel with the live animals and provide predictions of various scenarios, from further effects on health and welfare under different conditions and treatments, to optimizing mitigation scenarios (17, 178, 179).

## 7 Measuring complexity of empirical data

Measuring complexity is challenging since there is a variety of views on what complexity is that do not always coincide (97). The first (180) formulation is based on the information theory and Shannon entropy (181). Here complexity is based on patterns of statistical distribution, pointing to the degree of disorder, uncertainty and randomness. There are also several generalizations of Shannon entropy that can be especially useful for analyzing empirical time series data. For example, permutation entropy provides a measure of irregularity for time series (illustrated in Box 2) based on quantifying the relative ordering of neighboring values (164, 182, 183). Rényi entropy includes a parametric family of indices rather than a single value, thereby providing a more comprehensive description (183, 184). Randomness and unpredictability are also closely linked to the Brownian random walk, which can be described by the power law, in turn, allowing to propose further quantitative indices, such as the Hurst exponent (185). For example, a value of the Hurst exponent *H* = 0.5 indicates that the time series follows a classical random walk, *H* > 0.5 indicates that the time series is positively autocorrelated and potentially predictable, while *H* < 0.5 reveals a reversing, anti-persistent pattern.

Another approach is based on fractals: here the measure of complexity is given by the fractal dimension (186). This has been frequently used in physiology and neuroscience (187–190). Fractal dimension assesses geometric scale-dependency and persistent structural self-similarity. For instance, the multi-scale pattern of a structure space-filling could be simple and sparse (low fractal dimension) or dense and complex (high fractal dimension). Incidentally, Rényi entropy is directly connected to generalized fractal dimension (191). Most of the empirical studies discussed in the previous section used the fractal dimension for estimating complexity.

A different, although not unrelated (192), approach is based on algorithmic (Kolmogorov) complexity and algorithmic probability, which are uncomputable, although approximations exist (153, 156, 193). As discussed above, this view of complexity is framed in terms of the shortest program needed to generate a particular size of the output: a low Kolmogorov complexity ***K*** means that a particular data string requires a relatively short (simple) Turing machine to produce. Incidentally, an advantage of the Kolmogorov complexity over entropy is that the latter does not account for the specific patterns within the data and depends only on the statistical frequency distribution of the variable (signal). Yet, there is a link between Kolmogorov complexity and Shannon entropy (153) and small string approximations of Kolmogorov complexity behave like entropy in the worst case (158). Interestingly, algorithmic complexity of small Turing machines is strongly linked with their fractal dimension defined in terms of their output space–time diagram (192).

Another approach to assess complexity makes use of the largely empirically established Zipf’s law: the size distribution of entities is an inverse power law with a specific exponent (i.e., the number of entities with a size greater than *x* is inversely proportional to *x*). This relationship was initially discovered in linguistic as a link between word frequency vs. word rank and was explained by the principle of least effort (194), implicating an inverse link between the frequency and complexity. A lot of energy can be saved if frequently used linguistic units are shorter than those used more rarely (194–196). Zipf’s law describes a variety of phenomena: gene expression (197, 198), linguistics, cities, companies, human populations and economics (199–201). The power parameter of the relationship—Zipf exponent—therefore provides an indication of an overall scaling of entropy and its potential link with effort or energy required by the system. A larger Zipf exponent value (steeper relationship) implies higher predictability while a smaller value (a flat tail of the distribution) points to higher diversity. Incidentally, Shannon used the Zipf law to infer entropy of the English language (195). A theoretical explanation for Zipf law links objects ranking with diminishing probability to growing Kolmogorov complexity of the system (202–204).

Ladyman et al. (97) argue for statistical complexity that is based on quantifying the degree of organization rather than degree of uncertainty (randomness): the order presumably produced by a complex system (205, 206). A distinction of this approach is that an unpredictable random pattern is considered less rather than a more complex pattern. The unique assumption of the statistical complexity approach is that complex systems are not random, yet are also not ordered. Statistical complexity involves causal state reconstruction of a dynamic system. In simple words, this approach focuses on the degree of pattern rather than the degree of uncertainty. Further, the statistical complexity vs. nonparametric entropy curve has been found helpful to characterize the multifaceted nature of complex time series, such as distinguishing long- or short- range, oscillating, chaotic and stochastic processes (183, 207).

Finally, a very simple and practical measure of complexity, the roughness for a time series—von Neumann’s ratio of the mean square successive difference to variance (208)—requires sampling of only successive differences between the past and the present values. This suggests that simple organisms or even molecular automata could use it as an internal proxy for assessment of environmental complexity. Indeed, there is an indication that fish can respond to the environmental complexity measured this way (90).

This discussion is not exhaustive as determining the complexity and predictability of data, especially time series, raises a profound interest in many areas, such as economics, finances, neuroscience, climate research and so on. It is an active area of study with a several user-oriented reviews available (189, 190, 209–214). While previous studies of stress have used rather few approaches to measuring complexity—usually fractal dimension, but in some cases entropy (215) and Zipf exponent (171)—others exist. We suggest that empirical researchers will find it useful to distinguish different facets of complexity, such as uncertainty, algorithmic complexity and causal-organizational complexity. As such, statistical complexity and complexity-entropy analysis, we hope, will find a wider use.

Several R packages have been developed with focus on analyzing various complexity measures. For example, the ‘fractaldim’ packages contains various procedures to calculate fractal dimension (216). The ‘acss’ package (217) calculates algorithmic complexity using the block decomposition method for short strings (211). Furthermore, ‘statcomp’ package calculates various measures of statistical complexity and generalized entropy (218). Hurst exponent is available in the ‘pracma’ (219) and Zipf distribution, in ‘zipfR’ (220). The current availability of the major approaches for assessing the complexity of discrete and continuous data enables their wider use for stress detection and assessment.

## 8 Discussion

The association between stress and reduced behavioral complexity has long been documented, and its value as a highly sensitive, non-invasive indicator of animal welfare is well recognized. Measures of behavioral complexity can unveil effects of disturbances and stress that are not yet detectable using more conventional (e.g., level, degree, frequency) behavioral and physiological measures (167, 176, 221). The transition from behavioral control based on complex cognitive mechanisms to automated habitual (51, 60) is also consistent with the computational complexity view. However, a parsimonious and logically coherent theory explaining this relationship remained elusive. Here we propose a general control system level explanation that implicates the animal first-person computational and algorithmic complexity perspective. Stress is thought to occur when the controller program containing multiple input–output mappings fails to predict the environment and control behavior. Under such conditions, many inputs and feedback controls become increasingly obsolete and should be dynamically trimmed off along with the related modules, providing fitness benefit. Thus, computational complexity and intractability of adaptive control coupled with energy limitation are expected to reduce the size of the controller program and the complexity of its output, finally translating to the complexity of adaptive behavior.

This view is consistent with the existing theoretical frameworks for stress, involving functional reorganization, adaptive regulatory reprioritization and adaptive allocation of limited resources. Explicitly considering computational (in)tractability adds a new facet of understanding and explanation. All control processes are essentially computational. Therefore, uncertainty-induced regulatory intractability under stress is addressed by the organism through reallocation of its limited resources to processes that remain controllable and tractable. This requires functional reorganization and adaptive change of priorities to keep regulatory control as much as possible to maximize fitness, essentially making the best of a bad job. The computational complexity view of stress is therefore complementary to the traditional theoretical frameworks, however, it opens novel interesting questions for further formal studies. The computational perspective is also consistent with the Bayesian predictive paradigm. Computations required by the predictive probabilistic processing are extensive and energetically costly (58, 59, 222). Inability to predict and control environmental challenge and consequences of action could not be solved in a fitness-efficient way by unlimited scaling of the computational resources. On the other hand, many challenges result in depletion of the energetic resources and impair the computational capacity of the organism. Under these conditions, algorithmic complexity becomes the critical determinant of behavioral control. Transition to the emergency life-history stage can then be thought as an uttermost reduction of algorithmic complexity.

Based on the active inference paradigm and assuming any prediction error means stress, Goekoop and De Kleijn (50) argue that stress is expected to increase behavioral variability and complexity. This is thought to occur as a consequence of top-down collapse of the hierarchical goal system reducing the integrative control. We believe that this explanation is incoherent because it implicates both downregulation of computational resources, reducing contextual cues with simultaneous disinhibition and activation of strong, energetically (and computationally) expensive behavioral outputs required for increasing complexity. The evidence used by the authors [(50)] actually includes studies showing *reduction* of behavioral complexity (e.g., entropy (164), fractal-like properties (165)) under stress. We emphasize the distinction between challenge that results in a transient surprise and stress as the condition of gross unpredictability and uncontrollability. Transient surprise can result in defensive hormetic overcorrection and even increase behavioral complexity. This could solve the adaptive problem, facilitate learning, behavioral flexibility and calibrate further response control to analogous challenges. But the challenge could also escalate to uncontrollable stress state. According to the computational complexity view, it is the latter case that would result in reduction of behavioral complexity.

To examine the theoretical status of complexity view of stress one can use the Marr (223) framework (see Table 1) that has been remarkably fruitful in cognitive and computational neuroscience (224, 225). Marr argued that understanding any information processing system requires three levels of analysis: (1) computational theory, (2) representation and algorithm, (3) physical implementation. Then, the computational complexity view of stress outlined in this paper involves the abstract level 1 of understanding. It concerns the following questions: what is the end result of computation, its design goal (for biological systems, evolutionary design function), conceptual strategy and constraints. These questions concern the system manifestation of stress, the end result of mechanistic regulatory processes and the general phenomenological “design pattern” that is empirically observed. An important question concerns constraints and limitations: to what extent and under what conditions can the function—adaptive coping with challenge—be achieved, translating to computability and complexity. Further, the control systems perspective on stress implicating regulatory feedback and feed-forward mechanisms, putative proportional, integral and derivative controllers, solutions to regulatory trade-offs and similar mechanistic system architecture questions concern the algorithm-level explanation at the level 2. Finally, physiological mechanisms of stress including HPA axis, autonomic arousal, neurochemical and hormonal signaling, metabolic shifts and immune modulation represent the physical implementation (physiological realization) at the level 3. Following Marr, we posit that any exhaustive mechanistic understanding of a complex physiological phenomenon or system requires all three levels of analysis. For biological systems, the Marr’s levels of analysis correspond to the account for *mechanism* according to the Tinbergen’s four questions framework (226). Then, it should be complemented by answering the remaining Tinbergen’s questions: biological adaptive *function* (linked also with Marr’s level 1), *ontogeny* and *phylogeny.*

**Table 1:** Marr’s levels of mechanistic understanding: computation, algorithm, wet(hard)ware.

N	Level	Questions	Role in explanation of stress	Application to animal welfare
(1)	Computational theory	What is the goal, biological function, constraints and general strategy of the computation, mathematical formulation	Mathematical representation of complexity and challenge, resource limitation; computational complexity view of stress; approximate Bayesian inference minimizing variational free energy (mismatch between internal model and sensory data); principles of optimal control under resource constraint and resource reallocation	Theoretical principles; system-level detection
(2)	Representation and algorithm	How can the computational theory be implemented in terms of input, output and specific mechanistic processes, algorithms, how to represent the algorithm in a simulation model	Representation of regulatory feedback and feed-forward control systems, process-based algorithms accounting for mechanisms of stress appraisal, stress response, adjustment to uncertainty, resource reallocation	Mechanistic understanding, principles of prevention, mitigation and intervention, simulation models, digital twin systems
(3)	Biological realization	How the computational theory and the algorithm are realized in the biological system: biological processes, metabolism, hormonal and neural control, etc.	Specific physiological mechanisms of stress that realize the algorithms and mechanistic processes at the algorithm level. HPA axis, autonomic arousal, neurochemical and hormonal signaling, metabolic shifts, immune modulation etc.	Physiological detection and assessment of stress, developmental, environmental and medical interventions

## Data Availability

The original contributions presented in the study are included in the article/supplementary material, further inquiries can be directed to the corresponding author/s.

## Footnotes

1 Unlike the concept of “distress,” our view of “stress state” is not articulated as the consequence of a response, nor implicates subjective feeling or explicit detrimental consequences. The state of stress in this framework concerns specifically reduced capacity to perform adaptive control, making it also less vague than distress defined as “a state of the animal when it is stressed” (2).

## References

[1] Gregory NG. Physiology and Behaviour of Animal Suffering. Oxford: Blackwell Science (2004).

[2] Broom DM Johnson KG. Stress and animal Welfare. Key Issues in the Biology of Humans and other Animals. 2nd ed. Cham, Switzerland: Springer (2019).

[3] McCarty R. Stress, Health, and Behavior. New York: Guilford Publications (2023).

[4] Dawkins MS. Animal welfare as preventative medicine. Anim Welf. (2019) 28:137–41. doi: 10.7120/09627286.28.2.137

[5] Korte SM Koolhaas JM Wingfield JC McEwen BS. The Darwinian concept of stress: benefits of allostasis and costs of allostatic load and the trade-offs in health and disease. Neurosci Biobehav Rev. (2005) 29:3–38. doi: 10.1016/j.neubiorev.2004.08.009, 15652252

[6] Taborsky B English S Fawcett TW Kuijper B Leimar O McNamara JM . Towards an evolutionary theory of stress responses. Trends Ecol. Evol. (2021) 36:39–48. doi: 10.1016/j.tree.2020.09.003, 33032863

[7] Schreck CB Tort L. "The Concept of Stress in Fish". In: Schreck CB Tort L Farrell AP Brauner CJ, editors. Biology of Stress in Fish. Amsterdam: Academic Press (2016). p. 1–34. doi: 10.1016/B978-0-12-802728-8.00001-1

[8] Moberg GP. "Biological response to stress: implications for animal welfare". In: Moberg GP Mench JA, editors. The Biology of Animal Stress: Basic Principles and Implications for Animal Welfare. Wallingford: CABI (2001). p. 1–21.

[9] Koolhaas JM Bartolomucci A Buwalda B de Boer SF Flügge G Korte SM . Stress revisited: a critical evaluation of the stress concept. Neurosci Biobehav Rev. (2011) 35:1291–301. doi: 10.1016/j.neubiorev.2011.02.003, 21316391

[10] Peters A McEwen BS Friston K. Uncertainty and stress: why it causes diseases and how it is mastered by the brain. Prog Neurobiol. (2017) 156:164–88. doi: 10.1016/j.pneurobio.2017.05.004, 28576664

[11] Del Giudice M Buck CL Chaby LE Gormally BM Taff CC Thawley CJ . What is stress? A systems perspective. Integr Comp Biol. (2018) 58:1019–32. doi: 10.1093/icb/icy114, 30204874

[12] Bubic A Cramon DYV Schubotz RI. Prediction, cognition and the brain. Front Hum Neurosci. (2010) 4:1–15. doi: 10.3389/fnhum.2010.00025, 20631856 PMC2904053

[13] Clark A. Surfing Uncertainty: Prediction, action, and the Embodied mind. Oxford: Oxford University Press (2016).

[14] Deans C. Biological prescience: the role of anticipation in organismal processes. Front Physiol. (2021) 12:672457. doi: 10.3389/fphys.2021.672457, 34975512 PMC8719636

[15] Nakajima T. Biologically inspired information theory: adaptation through construction of external reality models by living systems. Prog Biophys Mol Biol. (2015) 119:634–48. doi: 10.1016/j.pbiomolbio.2015.07.008, 26196087

[16] Budaev S Jørgensen C Mangel M Eliassen S Giske J. Decision-making from the animal perspective: bridging ecology and subjective cognition. Front Ecol Evol. (2019) 7:164. doi: 10.3389/fevo.2019.00164

[17] Budaev S Kristiansen TS Giske J Eliassen S. Computational animal welfare: towards cognitive architecture models of animal sentience, emotion and wellbeing. R Soc Open Sci. (2020) 7:201886–6. doi: 10.1098/rsos.201886, 33489298 PMC7813262

[18] Mangel M. Modeling the animal in its world and the canonical problem for habitat selection. Isr J Ecol Evol. (2025) 71:1–19. doi: 10.1163/22244662-bja10116

[19] Maier SF Seligman MEP. Learned helplessness: theory and evidence. J Exp Psychol Gen. (1976) 105:3–46. doi: 10.1037/0096-3445.105.1.3

[20] Batsching S Wolf R Heisenberg M. Inescapable stress changes walking behavior in flies - learned helplessness revisited. PLoS One. (2016) 11:1–16. doi: 10.1371/journal.pone.0167066, 27875580 PMC5119826

[21] Madaro A Olsen RE Kristiansen TS Ebbesson LOE Nilsen TO Flik G . Stress in Atlantic salmon: response to unpredictable chronic stress. J Exp Biol. (2015) 218, 2538–2550. doi: 10.1242/jeb.120535 26056242

[22] Galluzzi L Yamazaki T Kroemer G. Linking cellular stress responses to systemic homeostasis. Nat Rev Mol Cell Biol. (2018) 19:731–45. doi: 10.1038/s41580-018-0068-0, 30305710

[23] Özbey NP Thompson MA Taylor RC. The regulation of animal behavior by cellular stress responses. Exp Cell Res. (2021) 405:112720. doi: 10.1016/j.yexcr.2021.112720, 34217715 PMC8363813

[24] Kerr R Jabbari S Johnston IG. Intracellular energy variability modulates cellular decision-making capacity. Sci Rep. (2019) 9:20196. doi: 10.1038/s41598-019-56587-5, 31882965 PMC6934696

[25] Kumar R Johnston IG. ATP dependence of decision-making capacity in a fine-grained model of gene regulatory networks. J R Soc Interface. (2024) 21:20240246. doi: 10.1098/rsif.2024.0246

[26] Bonora M Patergnani S Rimessi A De Marchi E Suski JM Bononi A . ATP synthesis and storage. Purinergic Signal. (2012) 8:343–57. doi: 10.1007/s11302-012-9305-8, 22528680 PMC3360099

[27] Anderson C Von Keyserlingk MAG Lidfors LM Weary DM. Anticipatory behaviour in animals: a critical review. Anim Welf. (2020) 29:231–8. doi: 10.7120/09627286.29.3.231

[28] Cerqueira M Millot S Felix A Silva T Oliveira GA Oliveira CCV . Cognitive appraisal in fish: stressor predictability modulates the physiological and neurobehavioural stress response in sea bass. Proc R Soc B Biol Sci. (2020) 287:20192922. doi: 10.1098/rspb.2019.2922, 32183629 PMC7126027

[29] Vindas MA Folkedal O Kristiansen TS Stien LH Braastad BO Mayer I . Omission of expected reward agitates Atlantic salmon (*Salmo salar*). Anim Cogn. (2012) 15:903–11. doi: 10.1007/s10071-012-0517-7, 22622814

[30] Vindas MA Johansen IB Vela-Avitua S Nørstrud KS Aalgaard M Braastad BO . Frustrative reward omission increases aggressive behaviour of inferior fighters. Proc R Soc B Biol Sci. (2014) 281:20140300. doi: 10.1098/rspb.2014.0300 PMC4043094 24759861

[31] Sterling P Eyer J. "Allostasis: a new paradigm to explain arousal pathology". In: Fisher S Reason J, editors. Handbook of Life Stress, Cognition and Health. Chichester: John Wiley & Sons (1988). p. 629–49.

[32] Schulkin J. Rethinking Homeostasis: Allostatic Regulation in Physiology and Pathophysiology. Cambridge, Massachusetts: Bradford Books (2003).

[33] McEwen BS Wingfield JC. What is in a name? Integrating homeostasis, allostasis and stress. Horm Behav. (2010) 57:105–11. doi: 10.1016/j.yhbeh.2009.09.011, 19786032 PMC2815096

[34] McEwen BS Bowles NP Gray JD Hill MN Hunter RG Karatsoreos IN . Mechanisms of stress in the brain. Nat Neurosci. (2015) 18:1353–63. doi: 10.1038/nn.4086, 26404710 PMC4933289

[35] Schulkin J Sterling P. Allostasis: a brain-centered, predictive mode of physiological regulation. Trends Neurosci. (2019) 42:740–52. doi: 10.1016/j.tins.2019.07.010, 31488322

[36] Goldstein DS McEwen B. Allostasis, homeostats, and the nature of stress. Stress. (2002) 5:55–8. doi: 10.1080/102538902900012345, 12171767

[37] McEwen BS Wingfield JC. The concept of allostasis in biology and biomedicine. Horm Behav. (2003) 43:2–15. doi: 10.1016/S0018-506X(02)00024-7, 12614627

[38] Sadoul B Vijayan MM. "Stress and growth". In: Biology of Stress in Fish. Amsterdam: Academic Press (2016). p. 167–205.

[39] Mousikou M Kyriakou A Skordis N. Stress and growth in children and adolescents. Horm Res Paediatr. (2023) 96:25–33. doi: 10.1159/000521074, 34814153

[40] Wingfield JC Maney DL Breuner CW Jacobs JD Lynn S Ramenofsky M . Ecological bases of hormone-behavior interactions: the emergency life history stage. Am Zool. (1998) 38:191–206. doi: 10.1093/icb/38.1.191

[41] Romero LM Dickens MJ Cyr NE. The reactive scope model - a new model integrating homeostasis, allostasis, and stress. Horm Behav. (2009) 55:375–89. doi: 10.1016/j.yhbeh.2008.12.009, 19470371

[42] Kotas ME Medzhitov R. Homeostasis, inflammation, and disease susceptibility. Cell. (2015) 160:816–27. doi: 10.1016/j.cell.2015.02.010, 25723161 PMC4369762

[43] Sterling P. What is Health? Allostasis and the Evolution of human design. Cambridge, MA: MIT Press (2020). 10.1002/ajhb.23698 34779544

[44] Clark A. Whatever next? Predictive brains, situated agents, and the future of cognitive science. Behav Brain Sci. (2013) 36:181–204. doi: 10.1017/S0140525X12000477, 23663408

[45] Friston KJ Rosch R Parr T Price C Bowman H. Deep temporal models and active inference. Neurosci Biobehav Rev. (2017) 77:388–402. doi: 10.1016/j.neubiorev.2017.04.009, 28416414 PMC5461873

[46] Parr T Friston KJ. Generalised free energy and active inference. Biol Cybern. (2019) 113:495–513. doi: 10.1007/s00422-019-00805-w, 31562544 PMC6848054

[47] Parr T Pezzulo G Friston KJ. Active Inference. The free Energy Principle in mind, brain, and Behavior. Cambridge, MA: MIT Press (2022).

[48] Kim CS. Free energy and inference in living systems. Interface Focus. (2023) 13:20220041. doi: 10.1098/rsfs.2022.0041, 37065269 PMC10102732

[49] Rescorla M. Bayesian Models of the mind. Cambridge: Cambridge University Press (2025).

[50] Goekoop R De Kleijn R. How higher goals are constructed and collapse under stress: a hierarchical Bayesian control systems perspective. Neurosci Biobehav Rev. (2021) 123:257–85. doi: 10.1016/j.neubiorev.2020.12.021, 33497783

[51] Arnaldo I Corcoran AW Friston KJ Ramstead MJD. Stress and its sequelae: an active inference account of the etiological pathway from allostatic overload to depression. Neurosci Biobehav Rev. (2022) 135:104590. doi: 10.1016/j.neubiorev.2022.104590, 35183594

[52] Ramstead MJD Badcock PB Friston KJ. Answering Schrödinger’s question: a free-energy formulation. Phys Life Rev. (2018) 24:1–16. doi: 10.1016/j.plrev.2017.09.001, 29029962 PMC5857288

[53] Mann SF Pain R Kirchhoff MD. Free energy: a user’s guide. Biol Philos. (2022) 37:33. doi: 10.1007/s10539-022-09864-z

[54] Torigoe M Islam T Kakinuma H Fung CCA Isomura T Shimazaki H . Zebrafish capable of generating future state prediction error show improved active avoidance behavior in virtual reality. Nat Commun. (2021) 12:5712. doi: 10.1038/s41467-021-26010-7, 34588436 PMC8481257

[55] Friston K Levin M Sengupta B Pezzulo G. Knowing one’s place: a free-energy approach to pattern regulation. J R Soc Interface. (2015) 12:20141383. doi: 10.1098/rsif.2014.1383, 25788538 PMC4387527

[56] Ishida T. A constructive chemical oscillator model demonstrates the emergence of homeostasis before genetic information through active inference. Discover Life. (2026) 56:2. doi: 10.1007/s11084-026-09723-x

[57] Friston K. Life as we know it. J R Soc Interface. (2013) 10:20130475. doi: 10.1098/rsif.2013.0475, 23825119 PMC3730701

[58] Kwisthout J Van Rooij I. Computational resource demands of a predictive Bayesian brain. Comput Brain Behav. (2020) 3:174–88. doi: 10.1007/s42113-019-00032-3

[59] Kwisthout J Wareham T Van Rooij I. Bayesian intractability is not an ailment that approximation can cure. Cogn Sci. (2011) 35:779–84. doi: 10.1111/j.1551-6709.2011.01182.x, 21609357

[60] Wirz L Bogdanov M Schwabe L. Habits under stress: mechanistic insights across different types of learning. Curr Opin Behav Sci. (2018) 20:9–16. doi: 10.1016/j.cobeha.2017.08.009

[61] Fischer S Ferlinc Z Hirschenhauser K Taborsky B Fusani L Tebbich S. Does the stress axis mediate behavioural flexibility in a social cichlid, *Neolamprologus pulcher*? Physiol Behav. (2024) 287:114694. doi: 10.1016/j.physbeh.2024.114694, 39260667

[62] Fontana BD Cleal M Gibbon AJ McBride SD Parker MO. The effects of two stressors on working memory and cognitive flexibility in zebrafish (*Danio rerio*): the protective role of D1/D5 agonist on stress responses. Neuropharmacology. (2021) 196:108681. doi: 10.1016/j.neuropharm.2021.108681, 34175323

[63] Goldstein DM. "Control theory applied to stress management". In: Hershberger WA, editor. Volitional action: Conation and Control. Amsterdam: North Holland (1989). p. 481–91.

[64] Jensen P Toates FM. Stress as a state of motivational systems. Appl Anim Behav Sci. (1997) 53:145–56. doi: 10.1016/S0168-1591(96)01156-2

[65] Toates F. Control of Behaviour. Berlin: Springer-Verlag (1998). p. 187.

[66] Albertos P. Feedback and Control for Everyone. Berlin, Heidelberg: Springer Berlin / Heidelberg (2010).

[67] Frank SA. Control Theory Tutorial: Basic Concepts Illustrated by Software Examples. Cham: Springer International Publishing (2018).

[68] Åström KJ Murray RM. Feedback Systems: an Introduction for Scientists and Engineers. 2nd ed. Princeton: Princeton University Press (2021). p. 505.

[69] Bhattacharya P Raman K Tangirala AK. Discovering adaptation-capable biological network structures using control-theoretic approaches. PLoS Comput Biol. (2022) 18:e1009769. doi: 10.1371/journal.pcbi.1009769, 35061660 PMC8809615

[70] Tkačik G Wolde PRT. Information processing in biochemical networks. Annu Rev Biophys. (2025) 54:249–74. doi: 10.1146/annurev-biophys-060524-102720, 39929539

[71] Powers WT. Behavior: the Control of Perception. 2nd ed. New Canaan, CT: Benchmark Publications (2005). p. 318.

[72] Csete ME Doyle JC. Reverse engineering of biological complexity. Science. (2002) 295:1664–9. doi: 10.1126/science.1069981, 11872830

[73] Frank SA. Evolutionary design of regulatory control. I. A robust control theory analysis of tradeoffs. J Theor Biol. (2019) 463:121–37. doi: 10.1016/j.jtbi.2018.12.023, 30571960

[74] Frank SA. Evolutionary design of regulatory control. II. Robust error-correcting feedback increases genetic and phenotypic variability. J Theor Biol. (2019) 468:72–81. doi: 10.1016/j.jtbi.2019.02.012, 30796941

[75] Frank SA. Robustness and complexity. Cell Syst. (2023) 14:1015–20. doi: 10.1016/j.cels.2023.11.003, 38128480

[76] Aoki SK Lillacci G Gupta A Baumschlager A Schweingruber D Khammash M. A universal biomolecular integral feedback controller for robust perfect adaptation. Nature. (2019) 570:533–7. doi: 10.1038/s41586-019-1321-1, 31217585

[77] Frei T Chang C-H Filo M Arampatzis A Khammash M. A genetic mammalian proportional–integral feedback control circuit for robust and precise gene regulation. Proc Natl Acad Sci. (2022) 119:e2122132119. doi: 10.1073/pnas.2122132119, 35687671 PMC9214505

[78] Filo M Chang C-H Khammash M. Biomolecular feedback controllers: from theory to applications. Curr Opin Biotechnol. (2023) 79:102882. doi: 10.1016/j.copbio.2022.102882, 36638743

[79] Howlett JR Paulus MP. Out of control: computational dynamic control dysfunction in stress- and anxiety-related disorders. Discov Ment Health. (2024) 4:5. doi: 10.1007/s44192-023-00058-x, 38236488 PMC10796870

[80] Nesse RM. The smoke detector principle. Natural selection and the regulation of defensive responses. Ann N Y Acad Sci. (2001) 935:75–85. doi: 10.1111/j.1749-6632.2001.tb03472.x, 11411177

[81] Haselton MG Nettle D. The paranoid optimist: an integrative evolutionary model of cognitive biases. Personal Soc Psychol Rev. (2006) 10:47–66. doi: 10.1207/s15327957pspr1001_3, 16430328

[82] Foster KR Kokko H. The evolution of superstitious and superstition-like behaviour. Proceed. R. Soc. Biol. Sci. (2008) 276:31–7. doi: 10.1098/rspb.2008.0981, 18782752 PMC2615824

[83] Johnson DDP Blumstein DT Fowler JH Haselton MG. The evolution of error: error management, cognitive constraints, and adaptive decision-making biases. Trends Ecol. Evol. (2013) 28:474–81. doi: 10.1016/j.tree.2013.05.014, 23787087

[84] Calabrese EJ Baldwin LA. Defining hormesis. Hum Exp Toxicol. (2002) 21:91–7. doi: 10.1191/0960327102ht217oa, 12102503

[85] Calabrese EJ. "Hormesis: what it is and why it matters". In: Mattson MP Calabrese EJ, editors. Totowa, NJ: Humana Press (2010). p. 1–13.

[86] Calabrese E. Hormesis: a fundamental concept in biology. Microbial Cell. (2014) 1:145–9. doi: 10.15698/mic2014.05.145, 28357236 PMC5354598

[87] Stebbing ARD. Growth hormesis: a by-product of control. Health Phys. (1987) 52:543–7. doi: 10.1097/00004032-198705000-00003, 2883157

[88] Baltieri M Buckley C. PID control as a process of active inference with linear generative models. Entropy. (2019) 21:257. doi: 10.3390/e21030257, 33266972 PMC7514737

[89] Ellis BJ Del Giudice M. Developmental adaptation to stress: an evolutionary perspective. Annu Rev Psychol. (2019) 70:111–39. doi: 10.1146/annurev-psych-122216-011732, 30125133

[90] Lai F Rønnestad I Budaev S Balseiro P Gelebart V Pedrosa C . Freshwater history influences farmed Atlantic salmon (*Salmo salar*) performance in seawater. Aquaculture. (2024) 586:740750. doi: 10.1016/j.aquaculture.2024.740750

[91] Vindas MA Madaro A Fraser TWK Höglund E Olsen RE Øverli Ø . Coping with a changing environment: the effects of early life stress. R Soc Open Sci. (2016) 3:160382. doi: 10.1098/rsos.160382, 27853554 PMC5098979

[92] Rachum A Harten LM Assa R Goldshtein A Chen X Gonceer N . Early experience affects foraging behavior of wild fruit bats more than their original behavioral predispositions. eLife. (2025) 14:RP103220. doi: 10.7554/eLife.103220, 41216964 PMC12604856

[93] Salvanes AGV Moberg O Ebbesson LOE Nilsen TO Jensen KH Braithwaite VA. Environmental enrichment promotes neural plasticity and cognitive ability in fish. Proc R Soc B Biol Sci. (2013) 280:20131331. doi: 10.1098/rspb.2013.1331, 23902903 PMC3735255

[94] Zhang Z Gao L Zhang X. Environmental enrichment increases aquatic animal welfare: a systematic review and meta-analysis. Rev Aquaculture. (2022) 14:1120–35. doi: 10.1111/raq.12641

[95] Kleiber A Stomp M Rouby M Ferreira VHB Bégout M-L Benhaïm D . Cognitive enrichment to increase fish welfare in aquaculture: a review. Aquaculture. (2023) 575:739654. doi: 10.1016/j.aquaculture.2023.739654

[96] Adami C. What is complexity? BioEssays. (2002) 24:1085–94. doi: 10.1002/bies.10192, 12447974

[97] Ladyman J Lambert J Wiesner K. What is a complex system? Eur J Philos Sci. (2013) 3:33–67. doi: 10.1007/s13194-012-0056-8

[98] Rebout N Lone J-C De Marco A Cozzolino R Lemasson A Thierry B. Measuring complexity in organisms and organizations. R Soc Open Sci. (2021) 8:200895. doi: 10.1098/rsos.200895 33959307 PMC8074971

[99] Badii R Politi A. Complexity: Hierarchical Structures and Scaling in Physics. Cambridge: Cambridge University Press (1997).

[100] Schuster P. How complexity originates: examples from history reveal additional roots to complexity. Complexity. (2016) 21:7–12. doi: 10.1002/cplx.21841, 41531421

[101] Lloyd S. Measures of complexity: a nonexhaustive list. IEEE Control Syst. (2001) 21:7–8. doi: 10.1109/MCS.2001.939938, 25079929

[102] Bray D. Wetware: A Computer in Every Living Cell. New Haven, CT: Yale University Press (2009). p. 1.

[103] Newman SA. "Complexity in organismal evolution". In: Philosophy of Complex Systems. Hooker CA, editor. Amsterdam: Elsevier (2011). p. 335–54.

[104] Kauffman SA. A World beyond Physics: The Emergence and Evolution of Life. New York: Oxford University Press (2019).

[105] Hochberg ME. An information framework of intelligence. Biosystems. (2025) 256:105548. doi: 10.1016/j.biosystems.2025.105548, 40789539

[106] Calvo P Baluška F. Conditions for minimal intelligence across eukaryota: a cognitive science perspective. Front Psychol. (2015) 6:1329. doi: 10.3389/fpsyg.2015.01329, 26388822 PMC4558474

[107] Pezzulo G Cisek P. Navigating the affordance landscape: feedback control as a process model of behavior and cognition. Trends Cogn Sci. (2016) 20:414–24. doi: 10.1016/j.tics.2016.03.013, 27118642

[108] Ginsburg S Jablonka E. The Evolution of the Sensitive soul. Learning and the Origins of Consciousness. Cambridge, MA: MIT Press (2019).

[109] Asher L Collins LM Ortiz-Pelaez A Drewe JA Nicol CJ Pfeiffer DU. Recent advances in the analysis of behavioural organization and interpretation as indicators of animal welfare. J R Soc Interface. (2009) 6:1103–19. doi: 10.1098/rsif.2009.0221, 19740922 PMC2817160

[110] Webber S Cobb ML Coe J. Welfare through competence: a framework for animal-centric technology design. Front. Vet. Sci. (2022) 9:885973. doi: 10.3389/fvets.2022.885973, 35847650 PMC9280685

[111] Arora S Barak B. Computational Complexity: A Modern Approach. Cambridge: Cambridge University Press (2009).

[112] Goldreich O. P, NP, and NP-Completeness: The Basics of Computational Complexity. Cambridge: Cambridge University Press (2010). p. 1.

[113] Davis M. The undecidable: basic papers on undecidable propositions, unsolvable problems, and computable functions. Mineola, NY: Dover Publication (2004).

[114] Reiter EE Johnson CM. Limits of Computation: an Introduction to the Undecidable and the Intractable. Boca Raton: CRC Press (2013).

[115] Cecconi F Cencini M Falcioni M Vulpiani A. Predicting the future from the past: an old problem from a modern perspective. Am J Phys. (2012) 80:1001–8. doi: 10.1119/1.4746070

[116] Lestas I Vinnicombe G Paulsson J. Fundamental limits on the suppression of molecular fluctuations. Nature. (2010) 467:174–8. doi: 10.1038/nature09333, 20829788 PMC2996232

[117] van Rooij I. The tractable cognition thesis. Cogn Sci. (2008) 32:939–84. doi: 10.1080/03640210801897856, 21585437

[118] van Rooij I Wright CD Wareham T. Intractability and the use of heuristics in psychological explanations. Synthese. (2012) 187:471–87. doi: 10.1007/s11229-010-9847-7

[119] Rich P Blokpoel M De Haan R van Rooij I. How intractability spans the cognitive and evolutionary levels of explanation. Top Cogn Sci. (2020) 12:1382–402. doi: 10.1111/tops.12506, 32500619 PMC7687229

[120] Xu J. Biological computing. Singapore: Springer Nature Singapore (2025).

[121] Prokopenko M Harré M Lizier J Boschetti F Peppas P Kauffman S. Self-referential basis of undecidable dynamics: from the liar paradox and the halting problem to the edge of chaos. Phys Life Rev. (2019) 31:134–56. doi: 10.1016/j.plrev.2018.12.003, 30655222

[122] Hart WE Istrail S. Robust proofs of NP-hardness for protein folding: general lattices and energy potentials. J Comput Biol. (1997) 4:1–22. doi: 10.1089/cmb.1997.4.1, 9109034

[123] Guyeux C Côté NM-L Bahi JM Bienia W. Is protein folding problem really a NP-complete one? First investigations. J Bioinforma Comput Biol. (2014) 12:1350017. doi: 10.1142/S0219720013500170, 24467756

[124] Karlebach G Shamir R. Modelling and analysis of gene regulatory networks. Nat Rev Mol Cell Biol. (2008) 9:770–80. doi: 10.1038/nrm2503, 18797474

[125] Karlebach G Robinson PN. Computing minimal Boolean models of gene regulatory networks. J Comput Biol. (2024) 31:117–27. doi: 10.1089/cmb.2023.0122, 37889991

[126] Wright AH Thompson RK Zhang J. The computational complexity of N-K fitness functions. IEEE Trans Evol Comput. (2000) 4:373–9. doi: 10.1109/4235.887236, 25079929

[127] Kaznatcheev A. Computational complexity as an ultimate constraint on evolution. Genetics. (2019) 212:245–65. doi: 10.1534/genetics.119.302000, 30833289 PMC6499524

[128] Tsotsos JK. Analyzing vision at the complexity level. Behav Brain Sci. (1990) 13:423–469. doi: 10.1017/S0140525X00079577

[129] Tsotsos JK. Behaviorist inteligence and the scaling problem. Artif Intell. (1995) 75:135–60. doi: 10.1016/0004-3702(94)00019-W

[130] Tsotsos JK. A Computational Perspective on Visual Attention. Cambridge, MA: MIT (2011). p. 333.

[131] Koechlin E. An evolutionary computational theory of prefrontal executive function in decision-making. Philos. Trans. R. Soc. B Biol. Sci. (2014) 369:20130474. doi: 10.1098/rstb.2013.0474, 25267817 PMC4186228

[132] Bossaerts P Murawski C. Computational complexity and human decision-making. Trends Cogn Sci. (2017) 21:917–29. doi: 10.1016/j.tics.2017.09.005, 29149998

[133] Levin M. Darwin’s agential materials: evolutionary implications of multiscale competency in developmental biology. Cell Mol Life Sci. (2023) 80:142. doi: 10.1007/s00018-023-04790-z, 37156924 PMC10167196

[134] Regev A Shapiro E. Cellular abstractions. Cells as computation. Nature. (2002) 419:343–3. doi: 10.1038/419343a, 12353013

[135] Brenner S. Life’s code script. Nature. (2012) 482:461–1. doi: 10.1038/482461a, 22358811

[136] Al-Hashimi HM. Turing, von Neumann, and the computational architecture of biological machines. Proc Natl Acad Sci. (2023) 120:e2220022120. doi: 10.1073/pnas.2220022120, 37307461 PMC10288622

[137] Pylyshyn ZW. Computation and Cognition. Cambridge, MA: MIT Press (1984).

[138] O’Reilly RC Munakata Y. Computational Explorations in Cognitive Neuroscience. Understanding the Mind by Simulating the Brain. Cambridge, MA: MIT Press (2000). p. 504.

[139] Brazma A. Living Computers: Replicators, Information Processing, and the Evolution of life. Oxford: Oxford University Press (2023).

[140] Colombo M Piccinini G. The Computational Theory of Mind. Cambridge: Cambridge University Press (2024).

[141] von Neumann J. First draft of a report on the EDVAC. IEEE Ann Hist Comput. (1993) 15:27–75. doi: 10.1109/85.238389

[142] Shannon CE. "A universal Turing machine with two internal states". In: NJA S Wyner AD, editors. Claude Elwood Shannon. Collected Papers. Piscataway, NJ: Wiley-Interscience (1993). p. 733–41.

[143] Rich E. Automata, Computability and Complexity: Theory and Applications. Upper Saddle River, N.J: Pearson Prentice Hall (2008). p. 1099.

[144] Moore C Mertens S. The Nature of Computation. Oxford: Oxford University Press (2019). p. 985.

[145] Kauffman SA. Investigations. Oxford: Oxford University Press (2000). p. 287.

[146] Dueñas-Díez M Pérez-Mercader J. How chemistry computes: language recognition by non-biochemical chemical automata. From finite automata to Turing machines. iScience. (2019) 19:514–26. doi: 10.1016/j.isci.2019.08.007, 31442667 PMC6710637

[147] Dueñas-Díez M Pérez-Mercader J. Native chemical computation. A generic application of oscillating chemistry illustrated with the Belousov-Zhabotinsky reaction. A review. Front Chem. (2021) 9:611120. doi: 10.3389/fchem.2021.611120, 34046394 PMC8144498

[148] Gorecki J. Information processing using networks of chemical oscillators. Entropy. (2022) 24:1054. doi: 10.3390/e24081054, 36010717 PMC9415872

[149] Piccinini G. Physical Computation: a Mechanistic Account. Oxford: Oxford University Press (2015). p. 1.

[150] Horsman D Stepney S Wagner RC Kendon V. When does a physical system compute? Proceed. R. Soc. Math. Physical Engin. Sci. (2014) 470:20140182. doi: 10.1098/rspa.2014.0182, 25197245 PMC4123767

[151] Horsman D. Abstraction/representation theory for heterotic physical computing. Philos Trans R Soc A Math Phys Eng Sci. (2015) 373:20140224. doi: 10.1098/rsta.2014.0224, 26078343

[152] Horsman D Kendon V Stepney S Young JPW. "Abstraction and representation in living organisms: when does a biological system compute?". In: Dodig-Crnkovic G Giovagnoli R, editors. Representation and Reality in Humans, other Living Organisms and Intelligent Machines. Cham: Springer International Publishing (2017). p. 91–116.

[153] Li M Vitányi P. An Introduction to Kolmogorov Complexity and its Applications. 4th ed. New York: Springer (2019).

[154] Kolmogorov AN. Three approaches to the quantitative definition of information ^*^. Int J Comput Math. (1968) 2:157–68. doi: 10.1080/00207166808803030

[155] Chaitin GJ. On the length of programs for computing finite binary sequences: statistical considerations. J ACM. (1969) 16:145–59. doi: 10.1145/321495.321506

[156] Zenil H. A review of methods for estimating algorithmic complexity: options, challenges, and new directions. Entropy. (2020) 22:1–28. doi: 10.3390/E22060612, 33286384 PMC7517143

[157] Zenil H Toscano FS Gauvrit N. Methods and Applications of Algorithmic Complexity: Beyond Statistical Lossless Compression. Berlin, Heidelberg: Springer (2022).

[158] Zenil H Hernández-Orozco S Kiani NA Soler-Toscano F Rueda-Toicen A Tegnér J. A decomposition method for global evaluation of Shannon entropy and local estimations of algorithmic complexity. Entropy. (2018) 20:1–46. doi: 10.3390/e20080605, 33265694 PMC7513128

[159] Dunbar RIM. Coevolution of neocortical size, group size and language in humans. Behav Brain Sci. (1993) 16:681–94. doi: 10.1017/S0140525X00032325

[160] Shettleworth SJ. Cognition, Evolution, and Behavior. 2nd ed. Oxford; New York: Oxford University Press (2010). p. 700.

[161] Dunbar RIM Shultz S. Social complexity and the fractal structure of group size in primate social evolution. Biol Rev. (2021) 96:1889–906. doi: 10.1111/brv.12730, 33945202

[162] Mason G Rushen J. Stereotypic animal Behaviour: Fundamentals and Applications to Welfare. 2nd ed. Wallingford: CABI (2006). p. 367.

[163] Deakin AG Spencer JW Cossins AR Young IS Sneddon LU. Welfare challenges influence the complexity of movement: fractal analysis of behaviour in zebrafish. Fishes. (2019) 4:1–16. doi: 10.3390/fishes4010008, 30654563

[164] Eguiraun H López-de-Ipiña K Martinez I. Application of entropy and fractal dimension analyses to the pattern recognition of contaminated fish responses in aquaculture. Entropy. (2014) 16:6133–51. doi: 10.3390/e16116133

[165] María GA Escós J Alados CL. Complexity of behavioural sequences and their relation to stress conditions in chickens (*Gallus gallus domesticus*): a non-invasive technique to evaluate animal welfare. Appl Anim Behav Sci. (2004) 86:93–104. doi: 10.1016/j.applanim.2003.11.012

[166] Alados CL Escos JM Emlen JM. Fractal structure of sequential behaviour patterns: an indicator of stress. Anim Behav. (1996) 51:437–43. doi: 10.1006/anbe.1996.0040

[167] Burgunder J Petrželková KJ Modrý D Kato A MacIntosh AJJ. Fractal measures in activity patterns: do gastrointestinal parasites affect the complexity of sheep behaviour? Appl Anim Behav Sci. (2018) 205:44–53. doi: 10.1016/j.applanim.2018.05.014

[168] Seuront L Cribb N. Fractal analysis reveals pernicious stress levels related to boat presence and type in the IndoPacific bottlenose dolphin, *Tursiops aduncus*. Phys A Stat Mech Appl. (2011) 390:2333–9. doi: 10.1016/j.physa.2011.02.015

[169] Cribb N Seuront L. Changes in the behavioural complexity of bottlenose dolphins along a gradient of anthropogenically-impacted environments in south Australian coastal waters: implications for conservation and management strategies. J Exp Mar Biol Ecol. (2016) 482:118–27. doi: 10.1016/j.jembe.2016.03.020

[170] Seuront L Cribb N. Fractal analysis provides new insights into the complexity of marine mammal behavior: a review, two methods, their application to diving and surfacing patterns, and their relevance to marine mammal welfare assessment. Mar Mamm Sci. (2017) 33:847–79. doi: 10.1111/mms.12399

[171] de Oliveira CGL Miranda JGV Japyassú HF El-Hani CN. Using Zipf–Mandelbrot law and graph theory to evaluate animal welfare. Phys A Stat Mech Appl. (2018) 492:285–95. doi: 10.1016/j.physa.2017.08.127

[172] MacIntosh AJJ Alados CL Huffman MA. Fractal analysis of behaviour in a wild primate: behavioural complexity in health and disease. J R Soc Interface. (2011) 8:1497–509. doi: 10.1098/rsif.2011.0049, 21429908 PMC3163426

[173] Alves LGA Winter PB Ferreira LN Brielmann RM Morimoto RI Amaral LAN. Long-range correlations and fractal dynamics in *C. elegans*: changes with aging and stress. Phys Rev E. (2017) 96:022417. doi: 10.1103/PhysRevE.96.022417, 28950588 PMC6011659

[174] Van De Leemput IA Wichers M Cramer AOJ Borsboom D Tuerlinckx F Kuppens P . Critical slowing down as early warning for the onset and termination of depression. Proc Natl Acad Sci USA. (2014) 111:87–92. doi: 10.1073/pnas.1312114110, 24324144 PMC3890822

[175] Ramesh A Nayak T Beestrum M Quer G Pandit J. Heart rate variability in psychiatric disorders: a systematic review. Neuropsychiatr Dis Treat. (2023) 19:2217–39. doi: 10.2147/NDT.S429592, 37881808 PMC10596135

[176] Raudies C Gygax L. The construction of a measure of behavioural complexity as a potential individual-based welfare indicator and its theoretical validation. Anim Welf. (2024) 33:e51. doi: 10.1017/awf.2024.48, 39703223 PMC11655276

[177] Cronin KA Ross SR. Technical contribution: a cautionary note on the use of behavioural diversity (H-index) in animal welfare science. Anim Welf. (2019) 28:157–64. doi: 10.7120/09627286.28.2.157

[178] Budaev S Dumitru ML Enberg K Handeland SO Higginson AD Kristiansen TS . Premises for a digital twin of the Atlantic salmon in its world: agency, robustness, subjectivity, and prediction. Aquacult. Fish Fish. (2024) 4:e153. doi: 10.1002/aff2.153

[179] Giske J Dumitru ML Enberg K Folkedal O Handeland SO Higginson AD . Premises for digital twins reporting on Atlantic salmon wellbeing. Behav Process. (2025) 226:105163. doi: 10.1016/j.beproc.2025.105163, 39909180

[180] Shannon CE. A mathematical theory of communication. Bell Syst Tech J. (1948) 27:379–423. doi: 10.1002/j.1538-7305.1948.tb01338.x

[181] Gray RM. Entropy and Information Theory. New York: Springer (2011).

[182] Bandt C Pompe B. Permutation entropy: a natural complexity measure for time series. Phys Rev Lett. (2002) 88:1–4. doi: 10.1103/PhysRevLett.88.174102, 12005759

[183] Jauregui M Zunino L Lenzi EK Mendes RS Ribeiro HV. Characterization of time series via Rényi complexity–entropy curves. Phys A Stat Mech Appl. (2018) 498:74–85. doi: 10.1016/j.physa.2018.01.026

[184] Guisande N Montani F. Rényi entropy-complexity causality space: a novel neurocomputational tool for detecting scale-free features in EEG/iEEG data. Front Comput Neurosci. (2024) 18:1342985. doi: 10.3389/fncom.2024.1342985, 39081659 PMC11287776

[185] Hurst HE. Long-term storage capacity of reservoirs. Trans Am Soc Civ Eng. (1951) 116:770–99. doi: 10.1061/TACEAT.0006518

[186] Seuront L. Fractals and Multifractals in Ecology and aquatic Science. Boca Raton, FL: CRC Press (2009).

[187] Paramanathan P Uthayakumar R. Application of fractal theory in analysis of human electroencephalographic signals. Comput Biol Med. (2008) 38:372–8. doi: 10.1016/j.compbiomed.2007.12.004, 18234169

[188] West BJ. Fractal physiology and the fractional calculus: a perspective. Front Physiol. (2010) 1:12. doi: 10.3389/fphys.2010.00012, 21423355 PMC3059975

[189] Leszczyński P Sokalski J. The use of fractal analysis in medicine: a literature review. Dent Med Probl. (2017) 54:79–83. doi: 10.17219/dmp/67501

[190] Meregalli V Alberti F Madan CR Meneguzzo P Miola A Trevisan N . Cortical complexity estimation using fractal dimension: a systematic review of the literature on clinical and nonclinical samples. Eur J Neurosci. (2022) 55:1547–83. doi: 10.1111/ejn.15631, 35229388 PMC9313853

[191] Zmeskal O Dzik P Vesely M. Entropy of fractal systems. Comput Math Appl. (2013) 66:135–46. doi: 10.1016/j.camwa.2013.01.017

[192] Joosten JJ Soler-Toscano F Zenil H. Fractal dimension versus process complexity. Adv Math Phys. (2016) 2016:5030593. doi: 10.1155/2016/5030593

[193] Zenil H Kiani NA Tegnér J. Methods of information theory and algorithmic complexity for network biology. Semin Cell Dev Biol. (2016) 51:32–43. doi: 10.1016/j.semcdb.2016.01.011, 26802516

[194] Zipf GK. Human Behaviour and the Principle of least Effort. New York: Hafner Publishing Company (1965).

[195] Muradyan R. "Scaling laws in complex systems". In: Arber W Mittelstrass J Sánchez Sorondo M, editors. Complexity and Analogy in Science: Theoretical, Methodological and Epistemological Aspects. Vatican City: Libreria Editrice Vaticana (2012). p. 106–11.

[196] Debowski L. Information Theory Meets power laws. Stochastic Processes and Language Models. Hoboken, NJ: John Wiley & Sons (2021).

[197] Kuznetsov VA Knott GD Bonner RF. General statistics of stochastic process of gene expression in eukaryotic cells. Genetics. (2002) 161:1321–32. doi: 10.1093/genetics/161.3.1321, 12136033 PMC1462190

[198] Furusawa C Kaneko K. Zipf’s law in gene expression. Phys Rev Lett. (2003) 90:088102. doi: 10.1103/PhysRevLett.90.088102, 12633463

[199] Newman M. Power laws, Pareto distributions and Zipf’s law. Contemp Phys. (2005) 46:323–51. doi: 10.1080/00107510500052444

[200] Corominas-Murtra B Solé RV. Universality of Zipf’s law. Phys Rev E. (2010) 82:011102. doi: 10.1103/PhysRevE.82.011102, 20866560

[201] Saichev A Malevergne Y Sornette D. Theory of Zipf’s law and beyond. Berlin, Heidelberg: Springer (2010).

[202] Manin YI. Zipf’s law and L. Levin probability distributions. Funct Anal Appl. (2014) 48:116–27. doi: 10.1007/s10688-014-0052-1

[203] Manin YI. Complexity vs energy: theory of computation and theoretical physics. J Phys Conf Ser. (2014) 532:012018. doi: 10.1088/1742-6596/532/1/012018, 42055534

[204] Alaskandarani M Dingle K. Low complexity, low probability patterns and consequences for algorithmic probability applications. Complexity. (2023) 2023:1–15. doi: 10.1155/2023/9696075, 42044246

[205] Crutchfield JP Young K. Inferring statistical complexity. Phys Rev Lett. (1989) 63:105–8. doi: 10.1103/PhysRevLett.63.105, 10040781

[206] Martin MT Plastino A Rosso OA. Generalized statistical complexity measures: geometrical and analytical properties. Phys A Stat Mech Appl. (2006) 369:439–62. doi: 10.1016/j.physa.2005.11.053

[207] Ribeiro HV Jauregui M Zunino L Lenzi EK. Characterizing time series via complexity-entropy curves. Phys Rev E. (2017) 95:062106. doi: 10.1103/PhysRevE.95.062106, 28709196

[208] von Neumann J. Distribution of the ratio of the mean square successive difference to the variance. Ann Math Stat. (1941) 12:367–95.

[209] Fulcher BD Little MA Jones NS. Highly comparative time-series analysis: the empirical structure of time series and their methods. J R Soc Interface. (2013) 10:20130048. doi: 10.1098/rsif.2013.0048, 23554344 PMC3645413

[210] Tang L Lv H Yang F Yu L. Complexity testing techniques for time series data: a comprehensive literature review. Chaos Solitons Fractals. (2015) 81:117–35. doi: 10.1016/j.chaos.2015.09.002

[211] Gauvrit N Singmann H Soler-Toscano F Zenil H. Algorithmic complexity for psychology: a user-friendly implementation of the coding theorem method. Behav Res Methods. (2016) 48:314–29. doi: 10.3758/s13428-015-0574-3, 25761393

[212] Raubitzek S Neubauer T. Combining measures of signal complexity and machine learning for time series analyis: a review. Entropy. (2021) 23:1672. doi: 10.3390/e23121672, 34945978 PMC8700684

[213] Lau ZJ Pham T Chen SHA Makowski D. Brain entropy, fractal dimensions and predictability: a review of complexity measures for EEG in healthy and neuropsychiatric populations. Eur J Neurosci. (2022) 56:5047–69. doi: 10.1111/ejn.15800, 35985344 PMC9826422

[214] Von Wegner F Wiemers M Hermann G Tödt I Tagliazucchi E Laufs H. Complexity measures for EEG microstate sequences: concepts and algorithms. Brain Topogr. (2024) 37:296–311. doi: 10.1007/s10548-023-01006-2, 37751054 PMC10884068

[215] Eguiraun H Lopez-De-Ipiña K Martinez I Discrimination of Contaminated fish Responses by Fractal Dimension and entropy Algorithms In 2014 International Work Conference on Bio-Inspired Intelligence: Intelligent Systems for Biodiversity Conservation, IWOBI 2014 – Proceedings. place: Liberia, Costa Rica. (2014) 173–177

[216] Gneiting T Ševčíková H Percival DB. Estimators of fractal dimension: assessing the roughness of time series and spatial data. Stat Sci. (2012) 247:277. doi: 10.1214/11-STS370, 24568719

[217] Gauvrit N. Singmann H. Toscano F. S. Zenil H. Acss: Algorithmic Complexity for Short Strings (2014) doi: 10.32614/CRAN.package.acss

[218] Sippel S. Lange H. Gans F. Statcomp: Statistical Complexity and Information Measures for Time Series Analysis (2019) doi: 10.32614/CRAN.package.statcomp

[219] Borchers H. W. Pracma: Practical Numerical math Functions (2011) doi: 10.32614/CRAN.package.pracma

[220] Evert S. Baroni M. zipfR: Statistical Models for Word Frequency Distributions (2006) doi: 10.32614/CRAN.package.zipfR

[221] Rutherford KMD Haskell MJ Glasbey C Lawrence AB. The responses of growing pigs to a chronic-intermittent stress treatment. Physiol Behav. (2006) 89:670–80. doi: 10.1016/j.physbeh.2006.08.006, 16982073

[222] Haueis P Colaço DJ. Metabolic considerations for cognitive modeling. Behav Brain Sci. (2025):1–53. doi: 10.1017/S0140525X25103956, 41249127

[223] Marr D. Vision. A Computational Investigation into the Human Representation and Processing of visual Information. Cambridge, MA: MIT Press (2010).

[224] Van Rooij I Baggio G. Theory before the test: how to build high-verisimilitude explanatory theories in psychological science. Perspect Psychol Sci. (2021) 16:682–97. doi: 10.1177/1745691620970604, 33404356 PMC8273840

[225] Krakauer JW Ghazanfar AA Gomez-Marin A MacIver MA Poeppel D. Neuroscience needs behavior: correcting a reductionist bias. Neuron. (2017) 93:480–90. doi: 10.1016/j.neuron.2016.12.041, 28182904

[226] Tinbergen N. On aims and methods of ethology. Z Tierpsychol. (1963) 20:410–33. doi: 10.1111/j.1439-0310.1963.tb01161.x

